# Differential expression and biochemical activity of the immune receptor Tim-3 in healthy and malignant human myeloid cells

**DOI:** 10.18632/oncotarget.5257

**Published:** 2015-09-16

**Authors:** Isabel Gonçalves Silva, Bernhard F. Gibbs, Marco Bardelli, Luca Varani, Vadim V. Sumbayev

**Affiliations:** ^1^ School of Pharmacy, University of Kent, Anson Building, Kent, ME4 4TB, United Kingdom; ^2^ Institute for Research in Biomedicine, Universita' della Svizzera italiana (USI) 6500 Bellinzona, Switzerland

**Keywords:** Tim-3, acute myeloid leukaemia, myeloid cells

## Abstract

The T cell immunoglobulin and mucin domain 3 (Tim-3) is a plasma membrane-associated receptor which is involved in a variety of biological responses in human immune cells. It is highly expressed in most acute myeloid leukaemia (AML) cells and therefore may serve as a possible target for AML therapy. However, its biochemical activities in primary human AML cells remain unclear. We therefore analysed the total expression and surface presence of the Tim-3 receptor in primary human AML blasts and healthy primary human leukocytes isolated from human blood. We found that Tim-3 expression was significantly higher in primary AML cells compared to primary healthy leukocytes. Tim-3 receptor molecules were distributed largely on the surface of primary AML cells, whereas in healthy leukocytes Tim-3 protein was mainly expressed intracellularly. In primary human AML blasts, both Tim-3 agonistic antibody and galectin-9 (a Tim-3 natural ligand) significantly upregulated mTOR pathway activity. This was in line with increased accumulation of hypoxia-inducible factor 1 alpha (HIF-1α) and secretion of VEGF and TNF-α. Similar results were obtained in primary human healthy leukocytes. Importantly, in both types of primary cells, Tim-3-mediated effects were compared with those induced by lipopolysaccharide (LPS) and stem cell factor (SCF). Tim-3 induced comparatively moderate responses in both AML cells and healthy leukocytes. However, Tim-3, like LPS, mediated the release of both TNF-α and VEGF, while SCF induced mostly VEGF secretion and did not upregulate TNF-α release.

## INTRODUCTION

The immune receptor T-cell immunoglobulin and mucin domain 3 (Tim-3) is involved in a variety of leukocyte biological responses [1]. In T cells it mainly mediates the inhibition of Th1 responses, while in dendritic cells it has a pro-inflammatory effect. In monocytes and macrophages Tim-3 is involved in phagocytosis [1]. However, in myeloid leukaemia cell lines Tim-3 was shown to induce a moderate growth factor/pro-inflammatory responses, similar to those observed by myeloid cell growth factors (for example stem cell factor – SCF) [2, 3].

Interestingly, Tim-3 is highly expressed in leukaemia cells, especially in T-cell lymphocytic leukaemia cells as well as acute myeloid leukaemia (AML) cells [4–6]. In leukaemic T cells Tim-3 agonists induce programmed cell death pathways, while in AML cells this does not seem to be the case [1, 6]. However, the biochemical mechanisms involved in the activation of human myeloid cells and, especially, in AML cells, are largely unknown.

Tim-3 is considered as one of the possible AML antigens, since malignant AML cells preserve the ability to express the SCF receptor (Kit or CD117) as well as Tim-3 [5, 7]. In contrast, myeloid stem cells only express Kit receptors but not Tim-3 [5]. Conversely, in most cases, mature myeloid cells express Tim-3 but not Kit receptors [5, 8]. Despite these differences in cell surface presence, total expression levels of Tim-3 in AML cells are also significantly increased compared to healthy leukocytes [5].

In myeloid cell lines and primary myeloid hematopoietic cells Tim-3 is known to mediate the activation of NF-kB transcription factor and TNF-α secretion [3]. Furthermore, in AML cell lines, Tim-3 moderately activates the mammalian target of rapamycin (mTOR, a master regulator of myeloid cell translational pathways). This results in activation of the hypoxia-inducible factor 1 (HIF-1) transcription complex, which upregulates glycolysis and expression/secretion of the pro-angiogenic vascular endothelial growth factor (VEGF) [2]. However, the differential effects of Tim-3 in *ex vivo* systems such as primary human AML cells versus healthy human leukocytes have not yet been elucidated.

In the present study, we therefore analysed both the total and cell surface expressions of the Tim-3 receptor in primary human AML blasts and healthy primary human leukocytes obtained from peripheral blood (buffy coats). We found that, in primary AML cells, Tim-3 expression is much higher compared to primary healthy leukocytes. We also observed that Tim-3 receptor molecules were mostly expressed on the surface of primary AML cells, while the majority of Tim-3 protein remained inside primary human healthy leukocytes. In primary human AML blasts (AML-PB001F), Tim-3 agonistic antibody as well as galectin-9 (one of the natural ligands of Tim-3) induced activation of the mTOR pathway (by mTOR-dependent phosphorylation of p70 S6 kinase 1 (p70 S6K1) and eIF4E-binding protein-1 (eIF4E-BP1)). This was in line with HIF-1 activation and increased secretion of VEGF and TNF-α. Similar results were obtained in primary human leukocytes isolated from buffy coats obtained from the blood of healthy donors. Importantly, in both types of primary cells, the effects were compared with those induced by lipopolysaccharide (LPS, a Gram-negative bacteria-derived toll-like receptor 4 (TLR4) ligand) and SCF (Kit ligand). In primary AML cells SCF induced the strongest biological response, whereas LPS displayed comparatively greater effects on primary human leukocytes. Tim-3 in both cases induced moderate cellular responses. However, although Tim-3, like LPS, triggered the release of both TNF-α and VEGF, SCF induced mostly VEGF secretion and did not significantly impact the TNF-α release.

## RESULTS

### Primary human AML blasts and healthy leukocytes express the Tim-3 immune receptor

Our recent work demonstrated that Tim-3 mediates the activation of mTOR *via* phosphorylation of its S2448 residue and HIF-1 signalling in human AML cell lines [2]. We therefore sought to understand the expression and behaviour of this receptor in primary human AML cells (AML-PB001F primary mononuclear blasts were used) in comparison with healthy whole blood primary human leukocytes (PLs).

**Figure 1:**
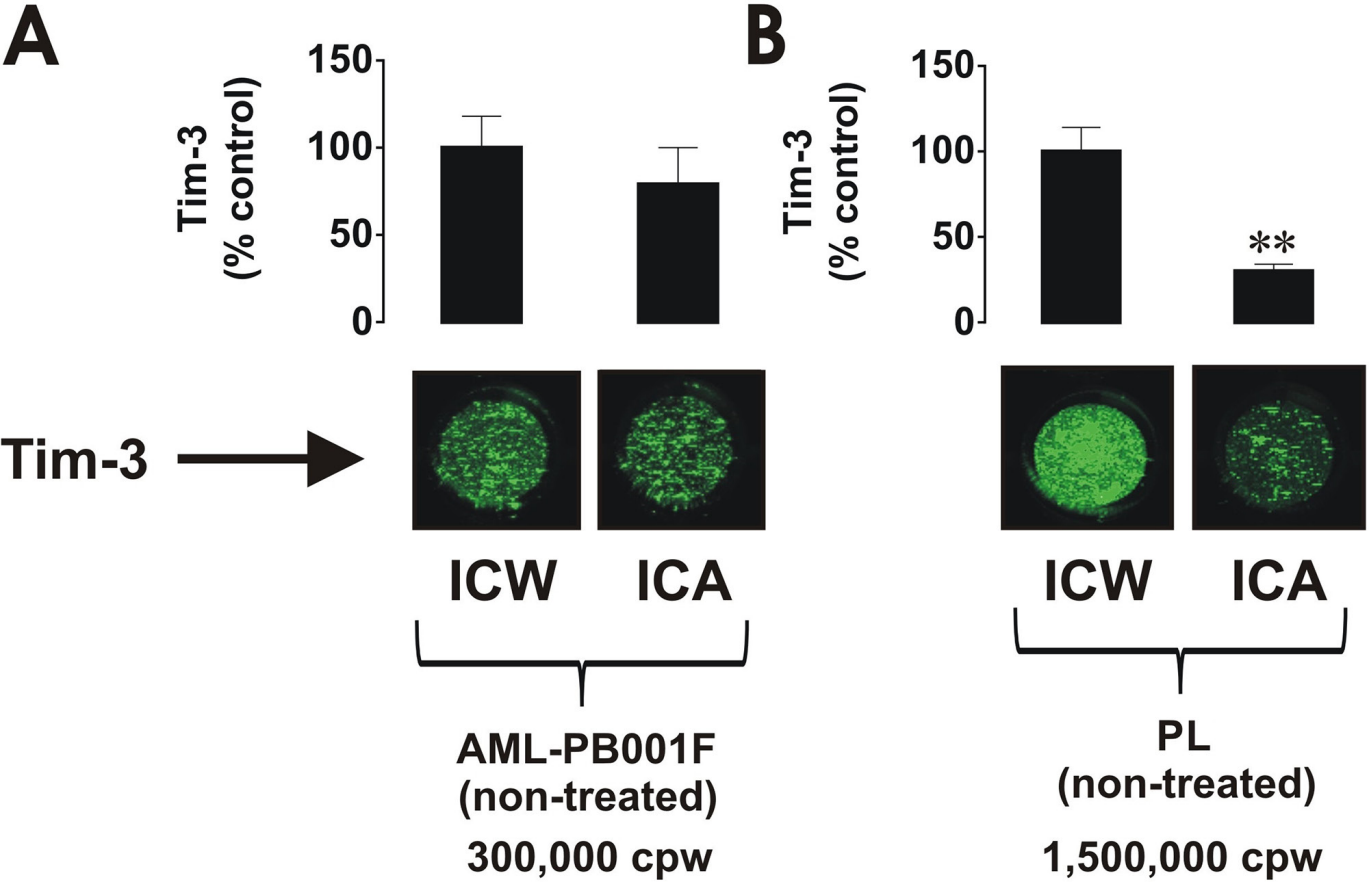
Comparative analysis of Tim-3 expression and surface presence in primary human AML cells and healthy leukocytes 300,000 per well of AML-PB001F primary human AML cells **A.** and 1,500,000 per well of healthy PLs **B.** were subjected to in-cell Western (ICW) in order to detect total Tim-3 expression. The Tim-3 surface presence was analysed by in-cell assay (ICA). For healthy PLs, 1,500,000 cells per well were necessary to properly visualise Tim-3 receptor on the cell surface. Fluorescence values obtained for AML cells and healthy PLs were divided by respective cell number (300,000 or 1,500,000) and used for calculations. Images are from one experiment representative of three which gave similar results. Quantitative data are shown as means ± SEM of at least three individual experiments; **p < 0.01 vs. control.

In order to compare the expression and, more importantly, re-distribution of Tim-3 in the cells we analysed its total amount and cell surface presence using in-cell Western and in-cell assay respectively. We found that both primary AML blasts and healthy whole blood leukocytes expressed Tim-3 as detected by in-cell Western and in-cell assay (Figure 1A and 1B). However, in AML cells most of the receptor molecules were externalised, whereas in healthy PLs only around 30% were present on the surface, clearly indicating that the vast majority of Tim-3 protein was stored inside the cell (Figure 1A and 1B), where Tim-3 function is unknown. These findings confirm that AML cells express more Tim-3 protein compared to healthy leukocytes and, importantly, AML cells retain almost all Tim-3 receptor molecules on their cell surface.

### Tim-3 triggers activation of the mTOR pathway and HIF-1 signalling in primary AML cells and primary healthy human leukocytes (PLs)

**Figure 2:**
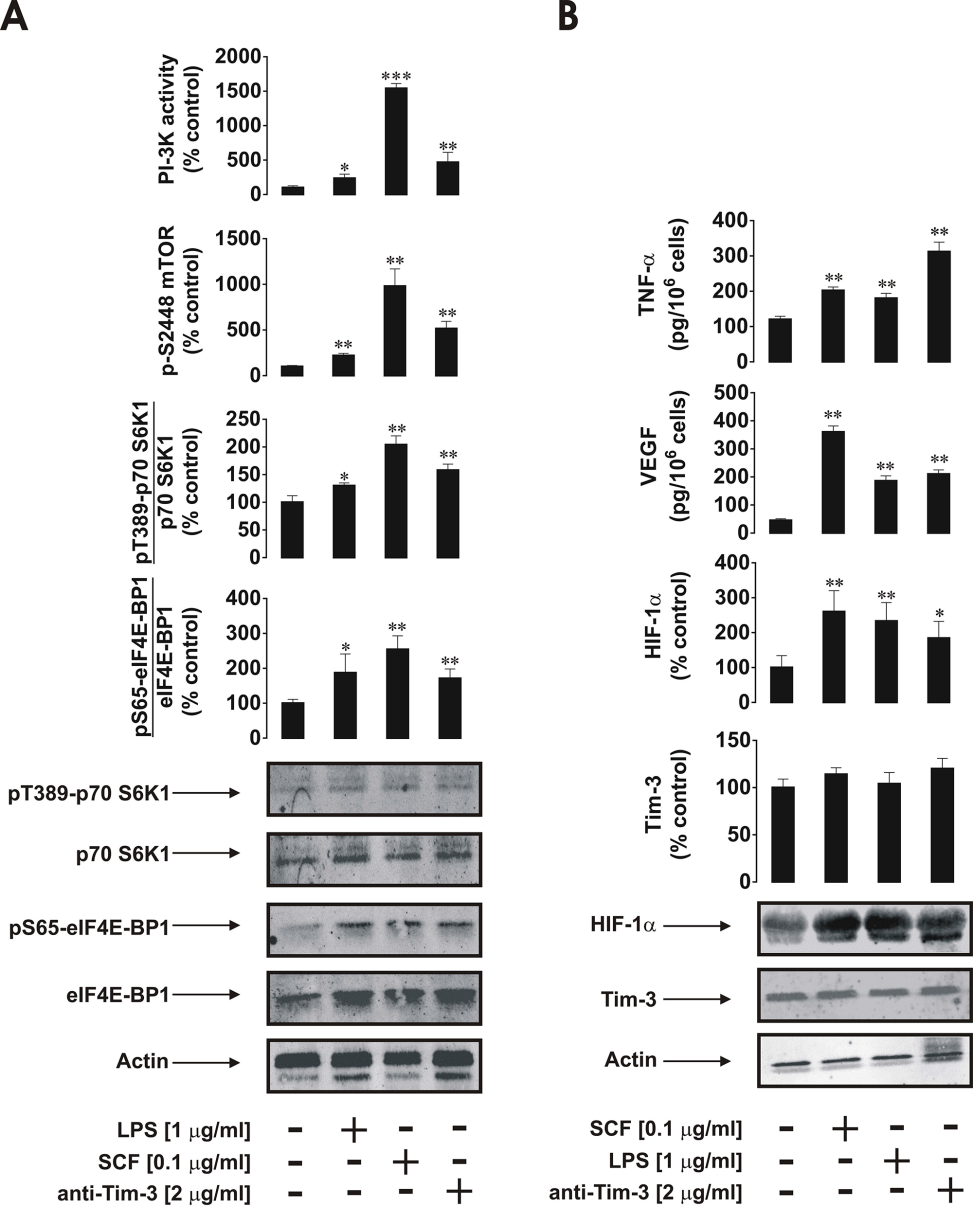
Anti-Tim-3 agonistic antibody induces mTOR activity, HIF-1α accumulation as well secretion of VEGF and TNF-α in primary human AML cells AML-PB001F cells were exposed for 4 h for the indicated concentrations of anti-Tim-3, LPS and SCF, followed by analysis of PI-3K/mTOR pathway activity **A.** HIF-1α accumulation and release of VEGF and TNF-α **B.** Images are from one experiment representative of four which gave similar results. Quantitative data are shown as means ± SEM of four individual experiments; *p < 0.05; **p < 0.01 vs. control.

Given the high expression/externalisation levels of Tim-3 protein in primary human AML cells compared to PLs we sought to obtain evidence on differences in receptor downstream activities. We found that, in primary AML cells, mTOR S2448 phosphorylation was substantially increased upon exposure to SCF as well as to anti-Tim-3 antibody stimulation (about 50% of SCF-induced effect), while LPS displayed a weaker ability to upregulate the intracellular levels of phospho-S2448 mTOR. The same pattern was observed for PI-3K activity (Figure 2A). Activity of LPS can be explained by the low TLR4 expression levels reported by the supplier for AML-PB001F primary human AML cells. These results were in line with mTOR activity levels monitored based on intracellular amounts of phospho-T389 p70 S6 K1 and phospho-S65 eIF4E-BP1 (Figure 2A).

Both proteins are phosphorylated at the indicated positions by the mTOR kinase complex [9]. All the stimuli demonstrated the ability to upregulate HIF-1α accumulation; anti-Tim-3 was the weakest inducer (Figure 2B) which is consistent with the observations made in cell lines. Stronger LPS-induced effects in this case were due to the fact that LPS-dependent HIF-1α accumulation, unlike the one triggered by both SCF and anti-Tim-3, did not solely depend on mTOR activation. LPS-induced TLR4-mediated HIF-1α accumulation is known to be jointly triggered through a redox-dependent mechanism, as well as mTOR and mitogen-activated protein (MAP) kinase signalling cascades [10, 11]. Therefore, the observed effects were achieved jointly *via* activation of all three pathways, the intensities of which were comparable with mTOR-dependent processes following exposure to SCF (Figure 2A and 2B). Total Tim-3 levels were not affected by any of the above stimuli, while in AML cell lines SCF and Tim-3 receptor activation upregulated total Tim-3 expression (Figure 2B). Importantly, as with LPS, anti-Tim-3 antibody was able to upregulate secretion of both TNF-α and VEGF, while SCF mostly upregulated VEGF production and did not dramatically change TNF-α secretion level (Figure 2B).

**Figure 3:**
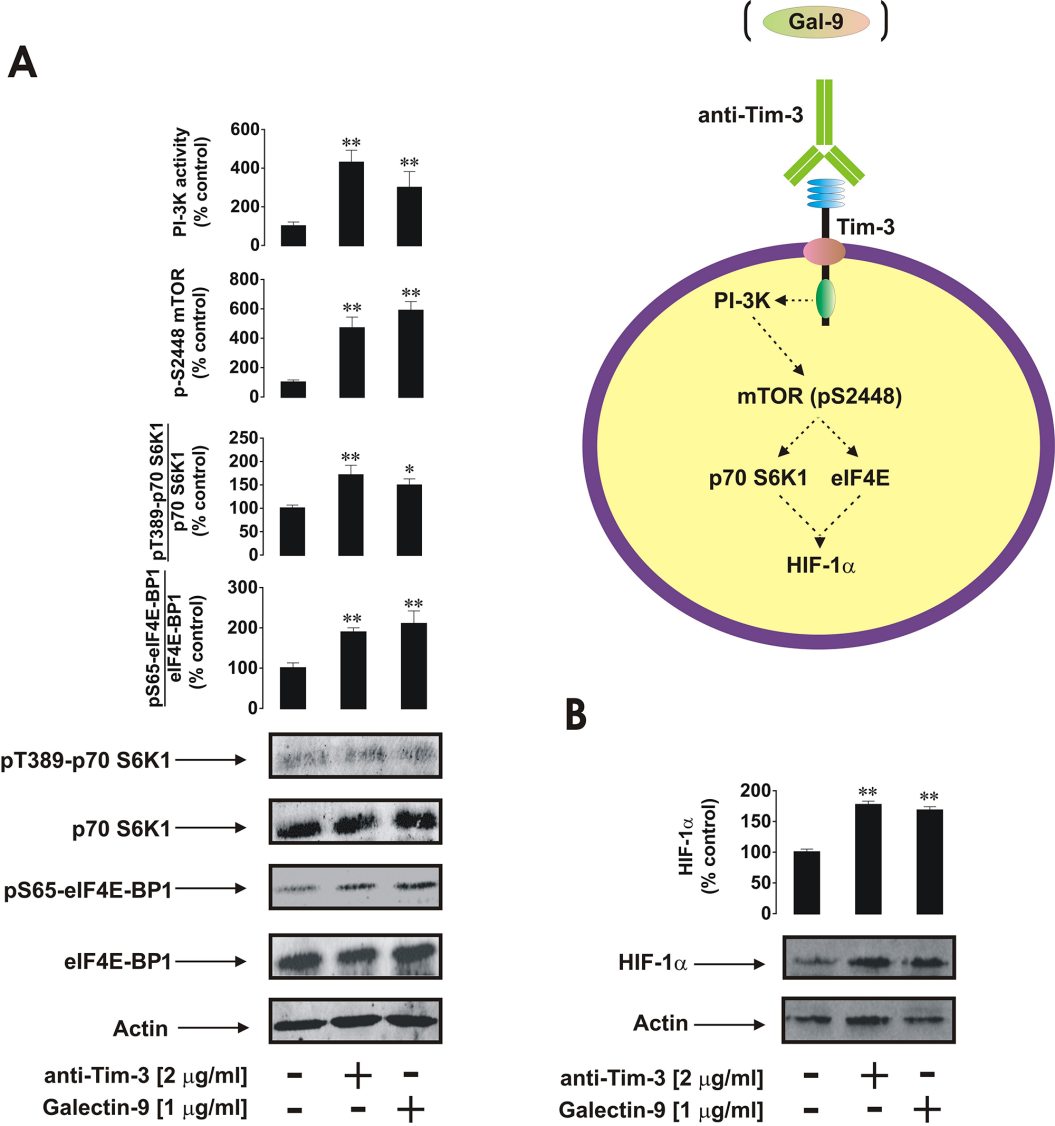
Anti-Tim-3 and galectin-9 induce similar responses in primary human AML cells AML-PB001F cells were exposed for 4 h for the indicated concentrations of anti-Tim-3 and galectin-9 followed by analysis of PI-3K/mTOR pathway activity **A.** and HIF-1α accumulation **B.** Images are from one experiment representative of three which gave similar results. Quantitative data are shown as means ± SEM of three individual experiments; *p < 0.05; **p < 0.01 vs. control.

Galectin-9, a natural Tim-3 ligand [2, 5], demonstrated the ability to upregulate both mTOR kinase activity and HIF-1α accumulation in primary AML-PB001F blasts at levels comparable to those seen with anti-Tim-3. This suggests similar activities of both agonistic ligands (Figure 3A and 3B).

**Figure 4:**
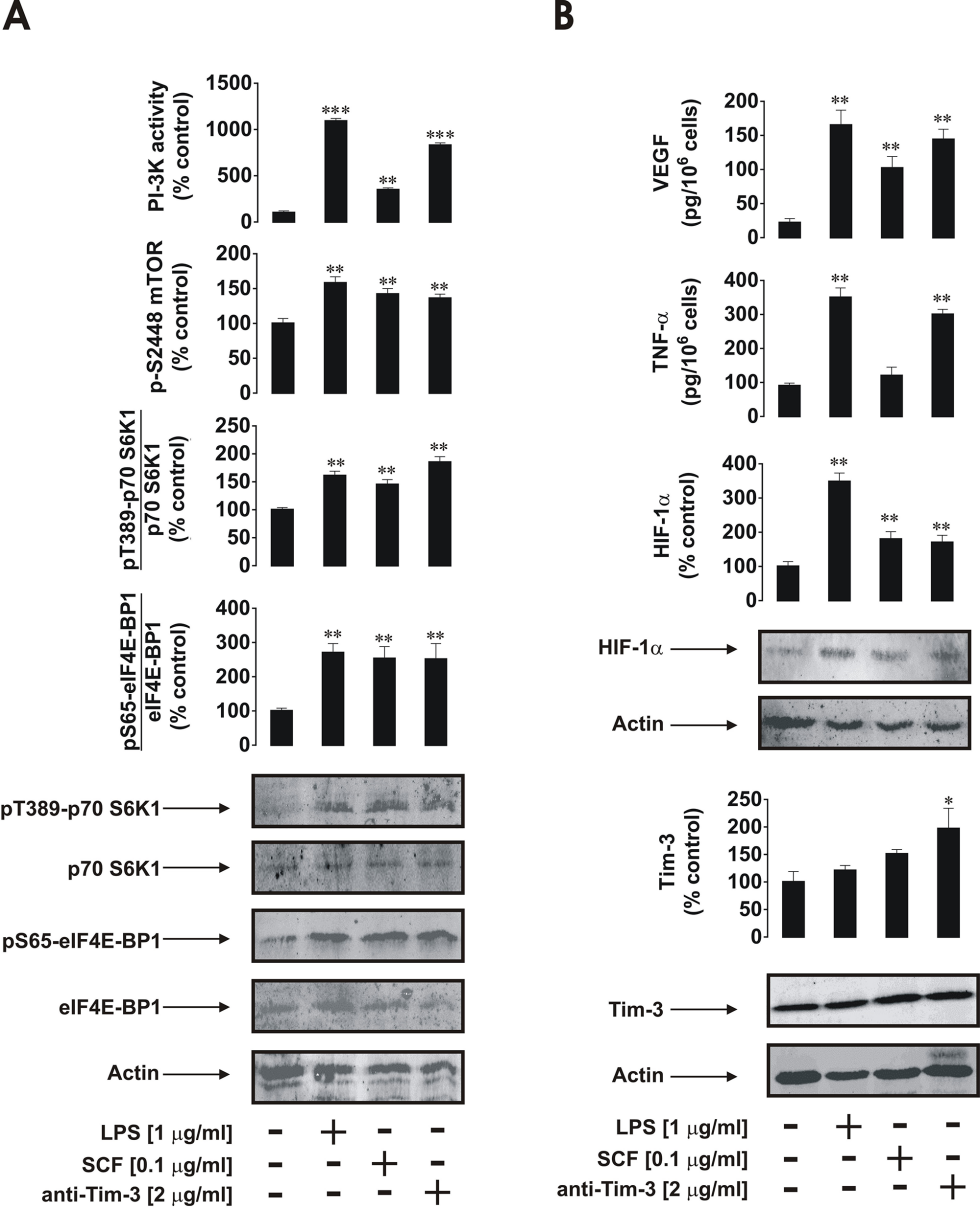
Anti-Tim-3 induces mTOR activity, HIF-1α accumulation as well secretion of VEGF and TNF-α in primary human healthy leukocytes PLs were exposed for 4 h for the indicated concentrations of anti-Tim-3, LPS and SCF followed by analysis of PI-3K/mTOR pathway activity **A.** HIF-1α accumulation and release of VEGF and TNF-α **B.** Images are from one experiment representative of four which gave similar results. Quantitative data are shown as means ± SEM of four individual experiments; *p < 0.05; **p < 0.01 vs. control.

In primary human healthy leukocytes the effects of anti-Tim-3 antibody were similar to those observed in AML cells. However, in healthy PLs LPS (and not SCF) was the most powerful cell activator (Figure 4A and 4B). This is probably due to the fact that healthy leukocytes contain more cells which express TLR4 than those that express Kit receptors. The effects of anti-Tim-3 antibody in this case were similar to those of SCF and slightly lower than the effects of anti-Tim-3 detected in primary human AML blasts (Figure 4A and 4B). Isotype control (IgG 2a) antibody [2] was also incubated with all the cell types studied and did not induce any effect in cell lines or primary leukocytes (data not shown).

**Figure 5:**
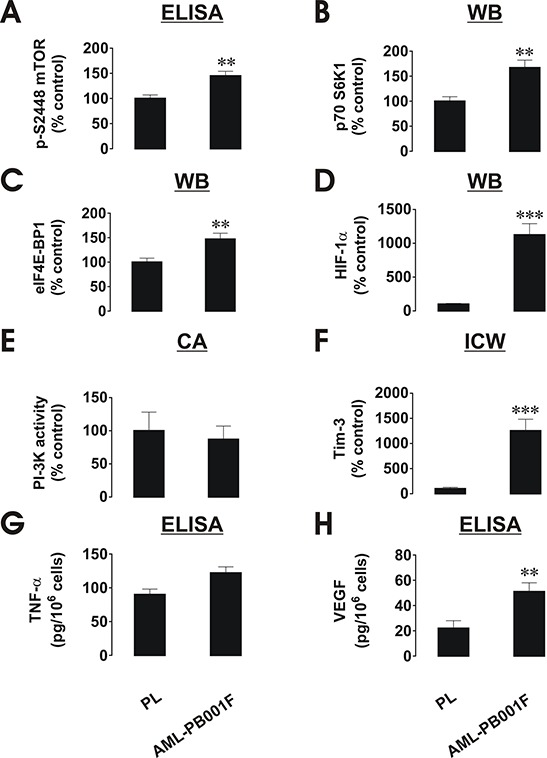
Comparative quantitative analysis of mTOR/HIF-1 pathway components as well as VEGF/TNF-α secretion in primary human AML cells and healthy human PLs Protein levels were compared based on the indicated type of detection. **A.** pS2448 mTOR intracellular levels were detected by ELISA, normalised against total cellular protein and compared (results were statistically validated). **B, C.** and **D.** – Levels of p70 S6K1, eIF4E-BP1 and HIF-1α protein were detected by Western blot (WB), quantitated and normalised against respective actin values before comparison and statistical validation of the results. **E.** PI-3K activity levels were measured by colorimetric assay (CA), **F.** – Intracellular levels of Tim-3 were detected by in-cell Westerns (ICW). Fluorescence values obtained for healthy PLs and AML cells and were divided by respective cell number (1,500,000 or 300,000, see Figure 1) and used for comparison (the value in PLs was considered as 100%). Similar results were obtained when comparing Tim-3 values in AML cells and healthy PLs obtained by Western blot and normalised against actin **G.** TNF-α and **H.** VEGF levels were measured in the medium by ELISA and the amounts per 10^6^ cells were compared and statistically validated. Data was obtained from 3–6 individual experiments, which gave similar results. Quantitative data are shown as means ± SEM; *p < 0.05; **p < 0.01; ***p < 0.01 vs. control.

Interestingly, background intracellular levels of phospho-S2448 mTOR, HIF-1α and Tim-3 proteins as well as VEGF, but not PI-3K activity and secreted TNF-α, were significantly higher in AML blasts compared to healthy PLs (a quantitative breakdown is shown in Figure 5A–5H). These findings confirm that the proteins discussed above are used by leukaemia cells on a permanent basis more than by healthy leukocytes.

## DISCUSSION

The immune receptor Tim-3 is expressed in healthy human hematopoietic cells and, despite numerous studies, its functional role is still a subject for intensive research [6]. Recent evidence demonstrated that the Tim-3 receptor is highly expressed and externalised in human AML cells [5, 7]. Importantly, progenitor hematopoietic cells do not express Tim-3 receptors while they produce substantial amounts of Kit (SCF) receptors. Mature healthy leukocytes generally lose their ability to express the Kit receptor but start expressing Tim-3 protein. Malignant AML cells express both receptors and thus their growth and proliferation depend on SCF while the biological activity of Tim-3 remains unclear [5]. It was reported that, in primary dendritic cells, Tim-3 induces TNF-α production through the NF-kB transcription factor [1, 3]. Our recent studies demonstrated that Tim-3 mediates activation of the PI3K/mTOR pathway in human AML cell lines [2]. However, it was not clear whether similar effects take place in primary human AML cells in comparison with healthy primary leukocytes, since activating both mTOR pathways and TNF-α production utilize both growth factor and inflammatory mediator-like responses.

We observed that primary AML cells generate more Tim-3 protein compared to healthy leukocytes, including cell surface protein expression. Only about 30% of Tim-3 molecules were externalised in primary healthy leukocytes, while almost all Tim-3 protein was present on the cell surface of primary AML cells. This could have impact on differences in activity of the protein in terms of its ability to mediate ligand-induced activation of mTOR, HIF-1α accumulation and cytokine releases. Our results suggest that Tim-3 mediates ligand-induced activation of the mTOR pathway, which involves upregulation of phospho-S2448 mTOR levels, phosphorylation of p70 S6K1(T389) and eIF4E-BP1 (S65), accumulation of HIF-1α protein and secretion of TNF-α and VEGF. It is however, clear that the effects are moderate and are weaker than those induced by LPS or SCF, a classic inflammatory mediator and hematopoietic growth factor, respectively.

In AML cells, SCF displayed the highest biological activity while LPS effects were stronger in healthy whole blood PLs. These differences are due to differential expression of TLR4 and Kit receptors in healthy and malignant leukocytes. SCF-induced responses in healthy PLs are likely to involve both direct and indirect effects. Several types of leukocytes express Kit receptors. This includes hematopoietic stem cells, multipotent progenitors, common myeloid progenitors, common lymphoid progenitors, megakaryocytes, myeloblasts, small lymphocytes, eosinophils, NK cells and dendritic cells [8, 12, 13]. Altogether, this amounts to more than 5–10% of blood leukocytes. SCF is known to trigger IL-6 release in primary cells [14] and we have also observed a clear increase in IL-6 secretion in healthy PLs exposed to 100 ng/ml SCF for 4 h (data not shown). IL-6 is known to upregulate VEGF release [15], which is a HIF-1-dependent process [16]. These effects could contribute to both HIF-1α accumulation and VEGF release in addition to SCF (primary stimulus, see Figure 4 for more details).

Although Tim-3 ligands are known to trigger programmed death of T cells and demonstrate downregulatory properties (especially malignant types, [1], 55–86% of all leukocytes are myeloid cells. Therefore, phenotypically, we observed the response of the vast majority of the total cell pool, indicating that the observed effects were likely to be derived from myeloid cells.

Intriguingly, the background levels of phospho-S2448 mTOR, HIF-1α, Tim-3 as well as p70 S6K1/eIF4E-BP-1 proteins are significantly higher in non-treated AML cells compared to resting healthy PLs (see Figure 5 for details). This phenomenon is especially applicable to the HIF-1α accumulation which is strong in non-treated AML cells and almost undetectable (detectable only upon loading around 140,000 cell per well) in healthy PLs. This observation becomes more intriguing in light of recently reported contribution of HIF-1 to Tim-3 expression [17]. Respectively, VEGF but not TNF-α secretions were significantly higher in non-treated AML cells compared to healthy PLs. Importantly, background levels of the PI-3K activity were very similar in AML cells and healthy PLs. This suggests that the increased background levels of pS2448 mTOR in primary AML cells could be due to increased constitutive phosphorylation as reported, for example, for the LAD2 mast cell line [18, 19]. LAD2 mast cells are also derived from the myeloid lineage, and were obtained from a 44-year-old male patient suffering from mastocytosis. Analysis of bone marrow biopsies/aspirates showed results consistent with an aggressive form of mast cell leukemia/sarcoma [20]. This suggests that the mTOR/HIF-1/VEGF pathway, but not inflammatory TNF-α, is used permanently by AML cells compared to healthy PLs.

**Figure 6:**
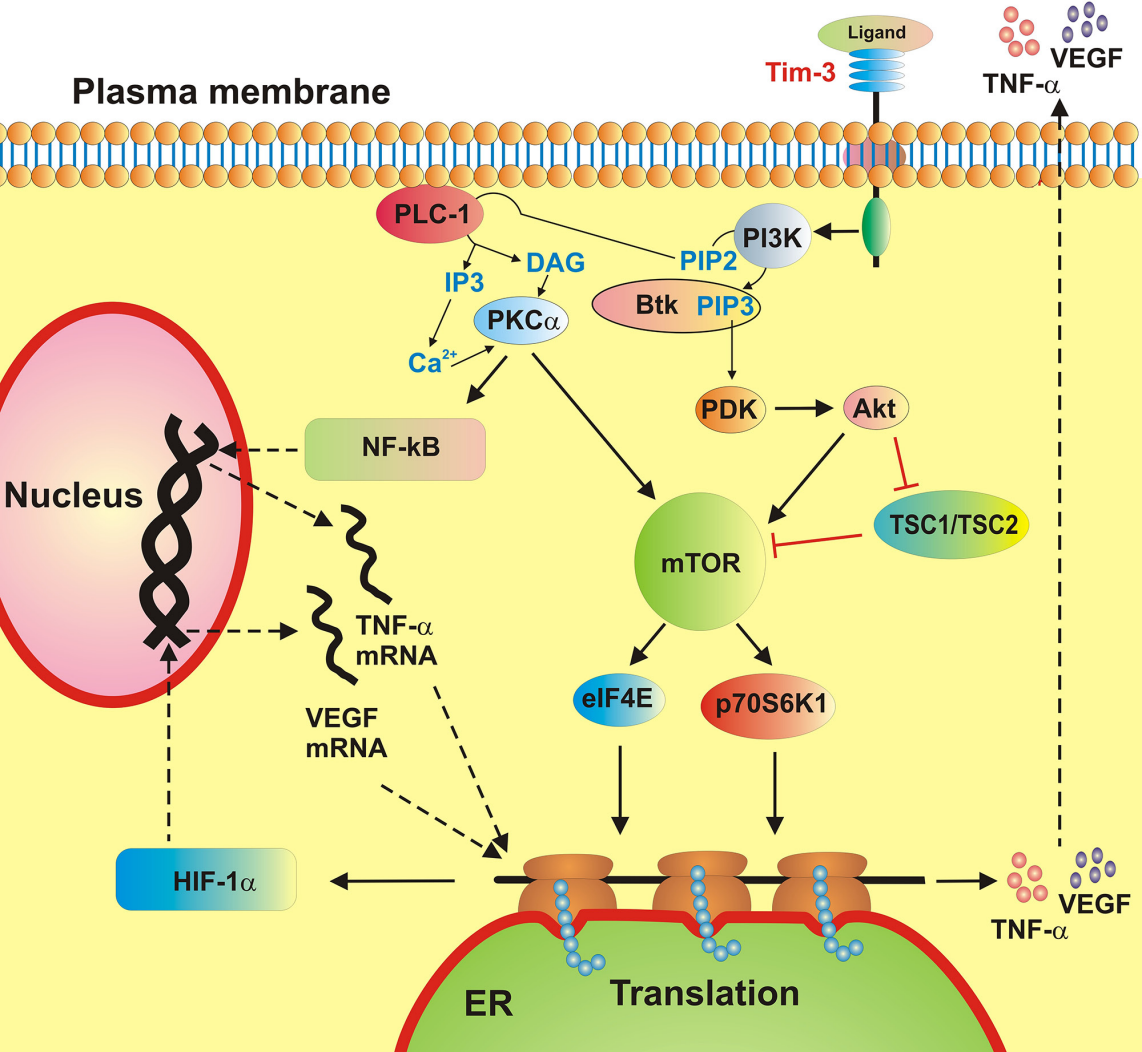
A schematic summary of ligand-induced Tim-3-mediated biological responses in healthy primary human leukocytes and primary human AML cells

Taken together, our findings indicate that Tim-3 in primary leukocytes, both healthy and malignant AML cells, displays moderate properties of an inflammatory receptor with additional growth factor (mTOR activation) and pro-angiogenic (VEGF release) activities (a summary of the biochemical activities of Tim-3 in AML cells is provided in Figure 6). Although the mTOR/HIF-1 pathway is triggered by both LPS and SCF, we only observed a significant increase in TNF-α/VEGF secretion (production of both factors is regulated by mTOR [21, 22]) following LPS stimulation. SCF induced mostly VEGF release and did not trigger the production of pro-inflammatory TNF-α. However, highly increased levels of Tim-3 protein in AML cells compared to those in healthy PLs did not result in higher specific biochemical activity of the receptor.

Currently, Tim-3 is being considered as a target antigen for anti-leukaemia therapy including T-cell leukaemia and AML [5] as well as to reverse T cell exhaustion and restore anti-tumour immunity [23]. In addition to the current knowledge, our findings suggest that Tim-3 is more abundant on the surface of AML cells compared to healthy leukocytes. This, together with the moderate biochemical activities associated with Tim-3 activation, is encouraging since it highlights a relatively easy way of recognition and selective targeting of human AML cells.

## MATERIALS AND METHODS

### Materials

RPMI-1640 medium, foetal calf serum and supplements, *P. aeruginosa* LPS, were purchased from Sigma (Suffolk, UK). Maxisorp™ microtitre plates were obtained either from Nunc (Roskilde, Denmark) or kindly provided by Oxley Hughes Ltd (London, UK). Mouse monoclonal antibodies to HIF-1α, mTOR and β-actin as well as rabbit polyclonal antibodies against phospho-S2448 mTOR were purchased from Abcam (Cambridge, UK). Antibodies against phospho-T389 p70 S6 kinase 1 (p70 S6K1), total and phospho-S65 eukaryotic initiation factor 4E binding protein 1 (eIF4E-BP1) antibodies were obtained from Cell Signaling Technology (Danvers, MA USA). Goat anti-mouse and goat anti-rabbit fluorescence dye-labelled antibodies were obtained from Li-Cor (Lincoln, Nebraska USA). ELISA-based assay kits for the detection of VEGF, TNF-α and IL-6 were purchased from Bio-Techne (R&D Systems, Abingdon, UK). All other chemicals were of the highest grade of purity and available commercially unless otherwise stated.

### Primary human AML cells

Primary human AML mononuclear blasts (AML-PB001F, newly diagnosed/untreated) were purchased from AllCells (Alameda, CA, USA) and handled in accordance with manufacturer's instructions. Cells from two different patients were analysed.

### Primary human leukocytes obtained from healthy donors (buffy coats)

Primary human leukocytes were obtained from buffy coat blood (which originated from healthy donors undergoing routine blood donation). The buffy coat blood was purchased from the National Health Blood and Transfusion Service (NHSBT, UK) following ethical approval (REC reference: 12/WM/0319). Mononuclear-rich leukocytes were obtained by Ficoll-density centrifugation according to the manufacturer's protocol. Cell numbers were determined using a haemocytometer and diluted accordingly with HEPES-buffered Tyrode's solution before treatment as indicated below.

### Stem cell factor

Human SCF protein was produced in *E.Coli* and purified in accordance with published protocols [24].

### Western blot analysis

Expressions of HIF-1α, total and phospho-T389 p70 S6K1, as well as total and phospho-S65 eIF4E-BP1 were determined by Western blot analysis and compared to β-actin in order to determine equal protein loading, as previously described [25]. Li-Cor goat secondary antibodies, conjugated with fluorescent dyes, were employed according to the manufacturer's protocol in order to visualise detected proteins (using a Li-Cor Odyssey imaging system). Following Western blot detection of phospho-T389 p70S6K1 and phospho-S65-eIF4E-BP1, membranes were stripped using a ReBlot™ Plus Kit (Chemicon International) according to the manufacturer's protocol. Membranes were then blocked and scanned using a Li-Cor Odyssey imaging system to make sure that stripping was successfully completed. Then membranes were re-probed with antibodies in order to detect the total amounts of p70S6K1 and eIF4E-BP1. Western blot data were quantitatively analysed using Odyssey software and values were normalised against respective β-actin bands.

### Detection of phospho-S2448 mTOR in cell lysates by ELISA

Phosphorylation of mTOR was analysed by ELISA as recently described [25]. Briefly, 96-well ELISA plates were coated with mouse anti-mTOR antibodies and then blocked with 2% BSA. Cell lysates were then added to the wells and incubated at room temperature for 2 h under constant agitation. After extensive washing with Tris-Buffered Saline and Tween 20 (TBST) buffer, anti-phospho-S2448 mTOR antibodies were added and plates incubated for 2 h at room temperature with constant agitation. After washing with TBST buffer plates were incubated with HRP-labelled goat anti-rabbit IgG (1:1000 dilution in TBST buffer). After 1 h incubation, plates were extensively washed using TBST and bound secondary antibodies were detected by the peroxidase reaction (ortho-phenylenediamine/H_2_O_2_).

### In-cell western and in-cell assay

We employed a standard Li-Cor in-cell Western assay to analyse total Tim-3 expressions in primary human AML cells and healthy primary leukocytes (PLs). The in-cell assay was also applied to detect Tim-3 protein surface presence in the studied cells [2].

### Detection of VEGF, TNF-α and IL-6 release

Concentrations of these cytokines released into the medium were analysed by ELISA (R&D Systems assay kits) according to the manufacturer's instructions.

### Analysis of phosphatidylinositol-3 kinase (PI-3K) activity

The activity of PI-3K was measured using a non-radioactive assay based on the ability of the enzyme to phosphorylate its substrate as previously described [26].

### Statistical analysis

Each experiment was performed at least three times and statistical analysis was conducted using a two-tailed Student's *t* test. Statistical probabilities (p) were expressed as *, where *p* < 0.05, **, where *p* < 0.01 and *** when *p* < 0.001.
